# P300 and Neuropsychological Assessment in Mild Cognitive Impairment and Alzheimer Dementia

**DOI:** 10.3389/fneur.2012.00172

**Published:** 2012-12-05

**Authors:** Mario A. Parra, Lindsay Lorena Ascencio, Hugo Fenando Urquina, Facundo Manes, Agustín M. Ibáñez

**Affiliations:** ^1^Scottish Dementia Clinical Research NetworkPerth, Scotland, UK; ^2^Human Cognitive Neuroscience, Psychology Department, University of EdinburghEdinburgh, UK; ^3^Neuropsy and Biomedical Unit, Health Faculty, Surcolombiana UniversityNeiva, Colombia; ^4^Laboratory of Experimental Psychology and Neurosciences, Institute of Cognitive Neurology and Institute of Neuroscience, Favaloro UniversityBuenos Aires, Argentina; ^5^Laboratory of Cognitive and Social Neuroscience, Universidad Diego PortalesSantiago, Chile; ^6^National Scientific and Technical Research CouncilBuenos Aires, Argentina

**Keywords:** Alzheimer’s disease, mild cognitive impairment, event related potentials, P300, neuropsychology, early detection, preclinical markers

## Abstract

Only a small proportion of individuals with Mild Cognitive Impairment (MCI) will convert to dementia. Methods currently available to identify risk for conversion do not combine enough sensitivity and specificity, which is even more problematic in low-educated populations. Current guidelines suggest the use of combined markers for dementia to enhance the prediction accuracy of assessment methods. The present study adhered to this proposal and investigated the sensitivity and specificity of the electrophysiological component P300 and standard neuropsychological tests to assess patients with Alzheimer’s disease (AD) and MCI recruited from a low-income country. The neuropsychological battery comprised tests of memory, attention, language, praxis, and executive functions. The P300 was recorded using a classical visual odd-ball paradigm. Three variables were found to achieve sensitivity and specificity values above 80% (Immediate and Delayed recall of word list – CERAD – and the latency of P300) for both MCI and AD. When they entered the model together (i.e., combined approach) the sensitivity for MCI increased to 96% and the specificity remained high (80%). Our preliminary findings suggest that the combined use of sensitive neuropsychological tasks and the analysis of the P300 may offer a very useful method for the preclinical assessment of AD, particularly in populations with low socioeconomic and educational levels. Our results provide a platform and justification to employ more resources to convert P300 and related parameters into a biological marker for AD.

## Introduction

According to Alzheimer’s Disease (AD) International, as many as 28 million of the world’s 36 million people with dementia have yet to receive a diagnosis, and therefore do not have access to treatment, information, and care (Alzheimer’s Disease International, 2011). They forecast that dementia will continue to affect the population worldwide and low-income countries will experience a more dramatic impact. A factor undermining the early diagnosis of dementia is the lack of reliable assessment methods (Sperling et al., 2011). The present study was aimed at investigating whether the combined use of neuropsychological and electrophysiological methods (i.e., P300) could help tackle this research priority. Particularly, this study investigated whether this combined approach would yield a methodology capable of achieving good classification powers (i.e., sensitivity and specificity) in populations with low socio-cultural background such as that found in Latin American countries (see Ardila et al., 1994).

Alzheimer’s disease is the most common form of dementia (Blennow et al., 2006). Although memory impairment is its most salient feature (Greene et al., 1996; Graham et al., 2004; Dudas et al., 2005; Nestor et al., 2006), the disease often presents with different neuropsychological phenotypes (Fields et al., 2011). This heterogeneity also characterizes the preclinical stages of AD [e.g., Mild Cognitive Impairment (MCI), Petersen, 2004, 2006; Petersen and Knopman, 2006; Petersen and Negash, 2008]. For example, different phenotypes of MCI have been identified and each has been associated with a different risk for AD (Dubois et al., 2007; Albert et al., 2011; Jack Jr. et al., 2011; Sperling et al., 2011). The study of individuals with MCI has shown that those who have memory impairment as a prominent feature in their cognitive profile (i.e., Amnestic MCI) have the highest probability of developing AD in the future (Bozoki et al., 2001; Lopez, 2003; Lopez et al., 2003; Petersen, 2006; Fields et al., 2011). Therefore, amnestic MCI is a preclinical form of dementia which can offer the best opportunity to investigate whether the combined use of neuropsychological tests and the P300 can aid in the early identification of changes suggestive of risk for AD. This was precisely the aim of the present study.

The presence of different biomarkers may suggest AD but do not lead to a definite diagnosis of AD (Albert et al., 2011; Jack Jr. et al., 2011; Sperling et al., 2011). Neuropsychological testing is crucial within this context. However, available memory tasks have not yet achieved sufficient diagnosis accuracy (i.e., combine sensitivity and specificity) as to grant them reliability in the detection of AD (Lowndes and Savage, 2007; Parra et al., 2010; Didic et al., 2011). Hence, there is a need for combined assessment tools which can improve the early diagnosis of dementia (Rachakonda et al., 2004; Dickerson et al., 2007; Dubois et al., 2007; Burns and Morris, 2008; Albert et al., 2011; Jack Jr. et al., 2011; Sperling et al., 2011). Event Related Potentials (ERPs), particularly the P300 wave, has proved to be sensitive to the early effects of AD (Muir et al., 1988; St Clair et al., 1988; Wright et al., 1988; Polich, 1989; Pokryszko-Dragan et al., 2003; Katada et al., 2004; Polich and Corey-Bloom, 2005; Ally et al., 2006; Bonanni et al., 2010; Lai et al., 2010). Using the classical odd-ball paradigm (Sutton et al., 1965) the characteristics of the P300 wave that have proved most useful in experimental and clinical settings are its amplitude and latency. These parameters are thought to be related to early conscious processes involved in attention and memory control (Donchin and Coles, 1988; Picton, 1992). As an index of early attentional and selection processes (i.e., a low-level cognitive function), the P300 component recorded during a classical odd-ball task does not seem to be reliant on the level of education of the assessed individual (see O’Donnell et al., 1995 for an example in schizophrenia). This makes it suitable to investigate cognitive decline in populations with low average education.

There is now sufficient evidence to suggest that the latency and amplitude of the P300 are altered in AD (Polich, 1989; Pokryszko-Dragan et al., 2003; Katada et al., 2004; Polich and Corey-Bloom, 2005; Ally et al., 2006; Muscoso et al., 2006; Caravaglios et al., 2008; Bonanni et al., 2010; Lai et al., 2010). Furthermore, evidence has been accrued suggesting that characteristics of the P300 wave are also compromised in individuals with MCI (Frodl et al., 2002; Golob et al., 2002; Bennys et al., 2007; van Deursen et al., 2009; Lai et al., 2010). Recent studies suggest that the latency and amplitude of the P300 wave might serve as a marker for monitoring the process through which MCI becomes AD (Golob et al., 2002, 2009; Papaliagkas et al., 2008; van Deursen et al., 2009). Changes in the P300 parameters have been identified in carriers of gene mutations that lead to familial AD almost 10 years before the disease onset (Golob et al., 2009). Taken together these results suggest that the P300 could contribute to the assessment of AD.

However, neither the P300 variables nor the neuropsychological tasks on their own have achieved enough specificity for a particular type of dementia such as AD (see for example Papaliagkas et al., 2008 for a report on poor correlations between P300 variables and other neuropsychological variables). More research is therefore needed to investigate whether the combined use of sensitive cognitive and biological markers can improve both the predictive and classification power of available assessment methods. Papaliagkas et al. (2010) combined the analysis of P300 with quantification of beta-amyloid (1–42) levels in Cerebrospinal Fluid (CSF). The authors reported values of sensitivity and specificity for the combination of CSF beta-amyloid levels and P300 latency of 80 and 98% respectively (100 and 89% for the P300 amplitude) in the discrimination between MCI converters and MCI stable patients. They suggested that the combination of electrophysiological and biological markers is a valid approach for the early diagnosis of AD. However, the analysis of the CSF requires an invasive procedure which can not be carried out outside health settings. Moreover, these assessment methods are not widely available in low-income countries. Computerized neuropsychological tests and portable systems for the recording of the P300 are now available. They are relatively inexpensive and can be used flexibly as to match patients’ environment (e.g., testing at home). Considering that P300 have been found to be sensitive even at very early stages of AD, this evidence warrants investigation of the subject addressed here.

Studies combining sensitive physiological and cognitive markers to investigate MCI are scarce. Only a handful of studies have used the analysis of the P300 component together with neuropsychological tasks to assess MCI and AD, and risk of MCI to AD conversion (Lastra et al., 2001; Lai et al., 2010; see also Revenok et al., 2001). These studies have focused on populations with a socio-cultural background very different to our own (Ardila et al., 1994), or have assessed groups of individuals with a non-specific risk for dementia (younger age bands, cortical, and subcortical dementia, etc.). Thus, the actual value of this combined approach for the early detection of AD still requires further investigation. The present study was aimed at investigating this issue in a sample of MCI patients who are known to be at a high risk for AD and in a sample of AD patients. Our prediction was that combining the analysis of the P300 (particularly P300 Latency, see Revenok et al., 2001 and Lai et al., 2010) with standard neuropsychological tests would yield more reliable outcomes in the identification of MCI and AD (i.e., increase sensitivity). We also predicted that the combined approach investigated here would also improve the specificity of the assessment process as the reliance of the P300 paradigm used in this study on the background education is minimal hence healthy controls who have limited cognitive reserve would be better classified (see Nitrini et al., 2009). We are not aware of previous studies which have addressed these issues with the methodology proposed here in the assessed population.

## Materials and Methods

The present study was reviewed and approved by the Ethics Committee of the Health Faculty at the Surcolombiana University, Colombia.

### Participants

A sample of 30 subjects was selected from the population studied by Gooding et al. (2006) following the procedures described below. Participants within each group (i.e., Healthy Controls, MCI, and AD) were randomly identified from our database. All the participants recruited into the study underwent a general interview, a neurological, and a neuropsychological examination. A multidisciplinary team including neurologists, psychiatrists, psychologists, and neuropsychologists performed the three assessment steps. When available, neuroimaging data also entered the diagnostic process. The team confirmed the diagnosis following the criteria set by NINCDS-ADRDA Group (McKhann et al., 1984) for AD and by Petersen (2004) for MCI. To be considered for the MCI group, participants should have subjective memory complaints with memory deficits documented by at least one objective memory test (minimum 1.5 SD below the norms). They should have no functional limitations as assessed by the Lawton Scale (see for example Morris, 2012 for recent suggestions). In addition to these criteria, participants were excluded from the study if they scored below 14 on the MMSE, had a previous history of psychiatric or neurologic disorders, were unable to consent by themselves, or presented with any kind of addiction or severe visual problems. The final sample comprised 10 patients with mild to moderate AD, 10 patients with MCI, and 10 healthy controls. All participants gave informed consent to take part in the study.

Table 1 presents the demographic and psychometric variables as well as the functional scales for the three groups, together with the result of statistical comparisons. For the comparison of these variables we used one-way ANOVA followed by Bonferroni-corrected *post hoc* tests. For all the comparisons alpha was set at 0.016 (three contrasts per each demographic variable).

**Table 1:** **Demographic, psychometric and functional variables in the selected sample**.

	Controls	MCI	AD	Controls vs. MCI	Controls vs. AD	MCI vs. AD
	(*n* = 10)	(*n* = 10)	(*n* = 10)	
	Mean (SD)	Mean (SD)	Mean (SD)	
Age	64.70 (4.24)	72.60 (8.11)	74.10 (5.72)	0.026	0.007	1.00
Education (years)	5.30 (4.03)	3.80 (4.39)	1.30 (1.83)	1.00	0.058	0.396
Gender (M/F)	3/7	4/6	4/6	0.85*		
MMSE	27.50 (2.95)	26.20 (2.30)	20.80 (4.37)	1.00	<0.001	0.003
GDS	1.10 (0.32)	2.10 (0.32)	2.80 (0.42)	<0.001	<0.001	<0.001
Depression (Yesavage)	1.20 (1.14)	3.20 (1.81)	3.40 (2.41)	0.070	0.041	1.00
IADL (Lawton)	8.00 (0.00)	9.40 (2.88)	12.00 (4.90)*	1.00	0.033	0.236

GDS, Global Deterioration Scale; IADL, Instrumental Activities of Daily Living; MMSE, Mini-Mental State Examination. *Post hoc* contrasts were significant at *p* < 0.016 (Bonferroni-corrected). *Chi-square revealed no significant differences in the sex by group distribution.

### Assessment

The assessment consisted of two parts, a neuropsychological battery and the analysis of the latency and amplitude of the P300. The neuropsychological assessment consisted of tests of Attention (Trail Making Test Part A and Letter A Cancelation including Hits and Time in seconds as the dependent variables for both tests), Memory (Memory for three Phrases, Word List including Immediate recall, Delayed recall and Recognition, and recall of the Complex Figure of Rey), Language (Phonological Fluency – Letter FAS and Boston naming test), Constructional Praxis (Copy of the Complex Figure of Rey), and Executive Functions (Wisconsin Card Sorting Test including Hits, Number of Categories and Conceptualization, and the Semantic Fluency Test – Animals). For a more detailed description of these tests and the Spanish norms see Ardila et al. (1994, 2000).

The ERP P300 component was recorded using the classic Odd-ball Paradigm with a visual version of the task. A two-channel DANTEC equipment (KeyPoint 1.0) that includes the P300 module was coupled with an external visual stimulator. The external stimulator was a standard computer running an application created add-hoc for the study. This application presented strings of 11 characters (e.g., XXXXXX) in the center of the screen. Each string was presented for 1 s. In 80% of the trials the characters were blue (distracter) while in the other 20% they were red (target). The participants were told to press a button only when the characters appeared in red color. To obtain the P300 component AgCl electrodes were placed at Fz (anterior) and Pz (posterior) according to the 10/20 international system. The impedances were kept below 10 kΩ. A total of 100 trials were presented which were averaged out to obtain the P300 wave. We calculated the peak latency of the P300 and the peak-to-peak amplitude and used them as the dependent variables.

### Statistical analysis

We performed a sample size calculation based on previous reports (Lai et al., 2010). Lai et al. (2010) reported that the latency of the P300 was the most sensitive variable in their analysis. In their follow up assessment MCI patients showed a latency of the P300 component in Pz of 466.77 (SD = 50.18) while controls showed a latency of 390.14 (SD = 27.23). This resulted in a large effect size (Cohen *d* = 1.9). Considering that in the present study we aimed at 80% of power with alpha set at 0.05 (critical *t* = 2.23), the number of participants required per group would be six. However, we aimed at a minimum of 10 participants per group as to control for variability particularly within the control group (considering the demographic characteristics of the assessed population such as low education).

For the analysis of the neuropsychological data we used one-way non-parametric Analysis of Variance (Kruskal–Wallis test) with the Group factor (Controls vs. MCI vs. AD) as the independent variable. Because age was found to be significantly different between AD patients and controls, and marginally different between Controls and MCI we used it as covariate. For the analysis of the latency and amplitude of the P300 component a mixed ANCOVA was used with Group (Controls vs. MCI vs. AD) as the between-subjects factor and Point (Fz vs. Pz) as the repeated measure. Following ANCOVA, Receiver Operating Characteristics (ROC) analysis was carried out to calculate the cut-off scores, the sensitivity and specificity, the Area under the Curve (AUC), and the 95% Confidence Interval for the neuropsychological and physiological variables (P300) that showed significant group effects.

## Results

### Neuropsychology

The results of the analysis of the neuropsychological data are shown in Table 2. Patients with MCI presented lower performance than controls in the following tasks: Recall of Word Lists, Semantic Verbal Fluency (animals), and the Wisconsin Card Sorting Test (Hits). However, when age-corrected comparisons were carried out, memory was the only neuropsychological function that remained significant across the two groups. This suggests that the MCI patients presented primarily with an amnesic deficit. Patients with AD showed lower performance than controls on the TMT, Letter A Cancelation (Hits), Immediate and Delayed recall of World Lists, Semantic Verbal Fluency Test (animals), Naming functions, Number of Categories reached in the WCST and the copy of the Complex Figure of Rey. These results support the multi-domain impairment of the AD group. Of note, AD and MCI patients did not significantly differ in the neuropsychological functions found to be impaired in the former group (with the exception of the TMT test and the copy and recall of the Complex Figure of Rey). This suggests that MCI patients’ performance on these tests fell between AD participants and controls (as reflected by the effect sizes). A combination of subthreshold impairment in these functions and a limited power due to the current sample size could explain these outcomes. Of note, this supports the early stage of AD of the recruited sample.

**Table 2:** **Performance of the three groups on the neuropsychological battery and results of the statistical analysis**.

	Mean (SD)			Kruskal–Wallis	Adjusted pairwise contrasts		
	Controls	MCI	AD	*p*-value	Controls vs. MCI	Controls vs. AD	MCI vs. AD
					*p*-value/*d*/β	*p*-value/*d*/β	*p*-value/*d*/β
TMT (hits)	23.90 (0.32)	22.70 (2.50)	12.00 (3.02)	0.001	ns/0.7/29	0.001/5.2/100	0.001/3.6/100
TMT (time s)	159.0 (60.01)	205.10 (93.54)	423.60 (172.48)	0.001	ns/0.5/23	0.001/2.0/99	0.001/1.6/91
Letter A cancelation (hits)	15.90 (0.32)	15.10 (1.10)	11.50 (4.60)	0.002	ns/1.0/55	0.001/1.5/81	ns/1.1/62
Letter A cancelation (time s)	78.20 (35.72)	71.90 (26.81)	76.10 (61.61)	0.889			
Memory for 3 phrases	2.70 (0.48)	2.30 (0.82)	2.30 (0.82)	0.437			
World list (immediate)	19.50 (3.21)	14.80 (2.66)	12.00 (4.83)	0.001	0.031/1.6/92	0.001/1.8/97	ns/0.7/33
World list (delayed)	7.10 (2.08)	5.10 (1.10)	2.70 (2.67)	0.003	ns/1.2/70	0.002/1.8/97	ns/1.2/70
World list (recognition)	19.40 (1.07)	19.00 (1.33)	17.00 (3.09)	0.066			
Verbal fluency (animals)	19.60 (3.86)	15.20 (3.36)	11.50 (3.92)	0.000	ns/0.3/11	0.001/1.03/60	ns/0.8/42
Verbal fluency (letters)	7.70 (4.37)	5.10 (3.67)	3.10 (3.75)	0.056			
Boston naming test	13.30 (1.77)	12.10 (1.60)	10.30 (1.57)	0.005	ns/0.7/32	0.004/1.7/96	ns/1.1/67
Rey figure (copy)	23.75 (9.76)	27.60 (7.75)	12.45 (6.55)	0.004	ns/0.4/15	0.01/1.4/82	0.001/2.1/99
Rey figure (recall)	10.00 (5.42)	13.45 (7.45)	4.65 (4.26)	0.008	ns/0.5/20	ns/1.1/64	0.007/1.4/86
WCST (hits)	19.80 (10.63)	14.40 (4.97)	13.20 (7.79)	0.211			
WCST (categories)	2.50 (1.72)	1.30 (0.67)	1.10 (9.83)	0.023	ns/0.6/71	0.033/0.7/84	ns/0.1/7
WCST (conceptualization)	11.60 (11.02)	16.90 (13.14)	15.7 (17.42)	0.318			

*Adjusted pairwise contrasts were carried out when the main effect of group was found to be significant*.

*Letter A Cancelation (Hits = number of letters correctly cancelled out of 16); TMT, Trial Making Test (Hits = number of circles correctly connected out of 25); WCST, Wisconsin Card Sorting Test (Hits = number of cards correctly classified out of 48, Short-version; Conceptualization = number of trials to first category); *p*-value/*d*/β = statistical significance/effect size (Cohen *d*)/Power (%) for the Bonferroni-corrected age-adjusted *post hoc* contrasts*.

### Event related potentials

**Figure 1:**
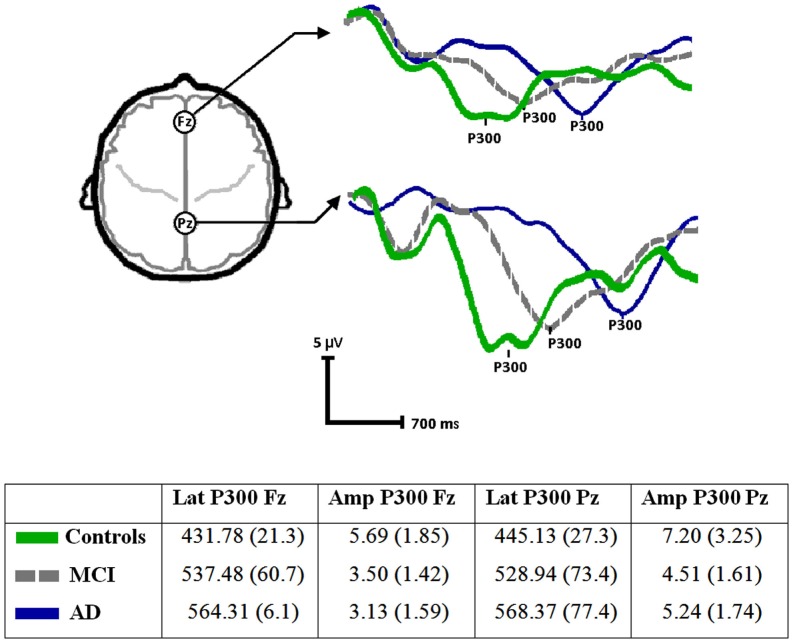
**ERPs (P300) waveforms and parameters (mean and SD) from the three groups (Controls, MCI, and AD) recorded at Fz (anterior) and Pz (posterior)**.

Figure 1 shows the average signals of the P300 component for the three groups as well as the descriptive statistics (mean and SD). Kolmogorov–Smirnov tests showed that the P300 variables were normally distributed (*p* = 0.2 for all). Hence, the validity of the model described above for the analysis of these variables was confirmed. The latency of the P300 component showed a significant effect of Group [*F*(2, 27) = 12.48, *p* < 0.001]. The Recording Point effect was found not to be significant (Pz vs. Fz) [*F*(1, 27) = 0.46, n.s.], nor was the Group by Recording Point interaction significant [*F*(2, 27) = 2.11, n.s.]. When age was entered as covariate, the effect of Group persisted [*F*(2, 27) = 9.96, *p* < 0.001]. *Post hoc* comparisons with Bonferroni corrections showed that patients with MCI and AD presented more prolonged latencies than controls (*p* < 0.05). The difference in the P300 latency between MCI and AD did not reach the threshold of significance.

The analysis of the amplitude of the P300 component using the statistical model described above showed a significant effect of Group [*F*(2, 27) = 4.63, *p* < 0.005] a significant effect of Recording Point [*F*(1, 27) = 18.91, *p* < 0.001] but no significant interaction between these factors [*F*(2, 27) = 0.65, n.s.]. *Post hoc* comparisons with Bonferroni corrections showed that the P300 amplitude in Fz was smaller in both MCI and AD patients than in healthy controls. No significant differences were found between groups in Pz.

The results presented above suggest that memory for word lists and both P300 parameters (Latency and Amplitude) could not only separate AD patients from healthy controls but they could also detect impairments in MCI patients at the group level. These results lend support to our hypothesis. However, to investigate whether this classification power also holds at the individual level, ROC analysis was carried out. For this analysis we chose performance on the Immediate and Delayed recall of Word Lists and both parameters of the P300 recorded in Fz (as no effect of the Recording Point was found in the latency analysis and the amplitude proved more sensitive at this site). We compared the AUC for the four measures and also calculated the sensitivity and specificity for each of them.

**Figure 2:**
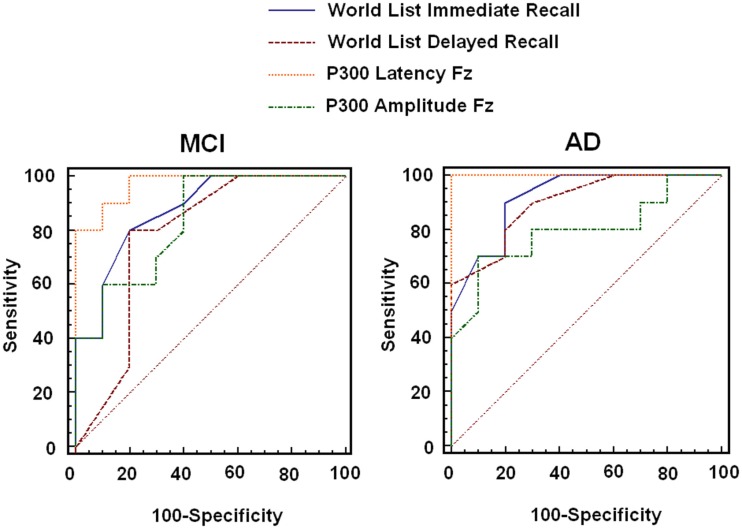
**Results from the ROC analysis carried out with neuropsychological and physiological variables that were found to be significant in group comparisons**.

As Figure 2 and Table 3 show, the latency of the P300 combined more sensitivity and specificity for MCI and AD than the other two memory tasks that also proved sensitive in this analysis and in previous ANCOVA. In fact, the latency of the P300 component proved to be the most sensitive measure. When the sensitivity and specificity were calculated based on the values of both the latency of the P300 and memory for word lists (combined sensitivity and specificity) using a series testing approach (which considers that both tests must be positive in order to prompt action, see Schoenbach and Rosamond, 2001), the sensitivity values for MCI increased considerably (96%) whereas the specificity remained high (80%). Of note a cut-of score >441.5 ms for the Latency of P300 resulted in a sensitivity of 100% and a specificity of 80% for MCI. This suggests that the combined use of neuropsychological and electrophysiological functions can offer better solutions for the detection of cognitive changes associated to MCI and AD.

**Table 3:** **Results of the ROC analysis with the variables which resulted in significant differences in group comparisons**.

	Controls vs. MCI						Controls vs. AD				
	Cut-off	AUC	SE	CI 95%	Sensitivity (%)	Specificity (%)	AUC	SE	CI 95%	Sensitivity (%)	Specificity (%)
Word list (immediate)	≤17	0.88	0.08	0.65–0.98	80	80	0.92	0.06	0.71–0.99	90	80
Word list (delayed)	≤5	0.78	0.12	0.54–0.93	80	80	0.90	0.07	0.68–0.99	80	80
Lat P300 FZ	>465.5	0.97	0.03	0.78–1.00	80	100	1.00	0.00	0.83–1.00	100	100
Amp P300 FZ	≤4.4	0.84	0.09	0.60–0.96	70	70	0.80	0.11	0.56–0.94	80	70

### Additional analysis

Finally, although this was not conceived as a longitudinal study, we approached our participants to reassess their neuropsychological functions. The initial assessment was concluded in 2005. From September 2011 to March 2012 (between 5 and 6 years after the first assessment) we were able to contact and reassess four patients initially seen as MCI and three healthy controls. Two of the initial MCI patients died in this interval, two had changed residence, one did not consent to take part in the reassessment, and one could not be contacted. Of the controls, one died, one did not consent to participate in the reassessment, and the others could not be contacted. We thought that although this dataset is limited it could still be informative. These subjects were reassessed using the same neuropsychological protocol and the criteria for MCI (Petersen, 2004) and AD (McKhann et al., 1984) were applied. Of the four MCI patients reassessed, two had converted to AD, one returned to normality, and one had an uncertain diagnosis. The patient with an uncertain diagnosis showed clear improvement in her global cognitive functions and other neuropsychological functions such as memory, attention, and executive function. However, her score on the IADL scale (Lawton) dropped relative to the first assessment. None of the healthy controls reassessed met MCI or AD criteria. The P300 data and the neuropsychological scores corresponding to the reassessed participants can be found in Table A1 in Appendix. The two MCI patients who converted to AD showed the longest latencies of the P300 component relative to the other reassessed participants. Of note, the MCI patient who returned to normality and the patient who received an uncertain diagnosis during the reassessment showed P300 latencies in the initial assessment which were within the normal limits.

## Discussion

The present study was set out to investigate whether the combined analysis of neuropsychological variables and variables of the P300 wave would yield classification powers (i.e., sensitivity and specificity) during the assessment of patients with MCI and AD better than those reported with each methodology separately. This hypothesis was investigated in a population with low socio-cultural background which is known to pose challenges to the interpretation of the outcomes of standard neuropsychological tasks (see Ardila et al., 1994). Three variables were found to achieve sensitivity and specificity values above 80% (Immediate and Delayed recall or word list – CERAD – and the latency of P300) for both MCI and AD. When they enter the model together (i.e., combined approach) the sensitivity for MCI increased to 96% and the specificity remained high (80%). These results lend support to our hypotheses. We now discuss the implications that these findings have for the assessment of MCI and AD.

The literature on AD and MCI reporting on the combined use of neuropsychological and psychophysiological tests is scarce. Lastra et al. (2001) reported findings similar to our own (see also Lai et al., 2010). These authors concluded that the latency of P300 is a useful tool in the early diagnosis of AD. This suggestion was supported by the observation of individuals with prolonged P300 latencies, who despite scoring 30 points on the MMSE, later developed AD. Recent findings of abnormal P300 parameters (i.e., long latencies and small amplitudes) in asymptomatic carriers of a gene mutation which leads to familial AD (Golob et al., 2009) almost 10 years before the disease onset support the validity of this test as a preclinical psychophysiological marker for AD. Neuropsychological tests and P300 variables have been used in combination for the evaluation of the therapeutic response to anticholinesterase drugs in patients with AD (Werber et al., 2001, 2003; Onofrj et al., 2002; Katada et al., 2003; Paci et al., 2006). However, the evidence provided by these earlier studies comes from rather heterogeneous (i.e., wide age ranges, different forms of dementia) and non-representative samples (e.g., with a level of education much higher than that observed in Latin American countries). The present study focused on a relatively small but more homogenous sample of amnestic MCI patients whose age was closer to that known to be associated with late-onset sporadic AD and whose education truly reflects the level reached by individuals of this age band in Latin American countries. This evidence is lacking in the literature concerning early detection of AD (Doraiswamy et al., 1995; Hong et al., 2011; see also Ardila et al., 1994).

One other study which is relevant to this discussion is one carried out by Lai et al. (2010). They also investigated the value of combining the study of the P300 with neuropsychological variables in patients with AD and MCI. They reported results similar to ours in a relatively larger group of patients. It is worth noting that the average education of their patients was 7.15 (5.03) and 9.89 (5.15) for AD and MCI respectively. This is much higher that the average education of our patients. However, the outcomes from both studies are similar. It is known that performance on traditional neuropsychological tests is highly sensitive to the subject’s educational level. For example in the present study the average MMSE value for the controls was 27.5. Other studies have observed this score in MCI patients. This may reflect, to a large extent, the low educational level of the population assessed in the present study and the sensitivity of adapted tests to this factor. Therefore, the results presented here suggest that the use of the P300 in the assessment of AD could overcome one of the most challenging issues in neuropsychological testing settings; that is, the cultural background of the assessed population (Ardila et al., 1994).

The electrophysiological component P300 recorded with the odd-ball paradigm presented here appears to be insensitive to trans-cultural variations. For example, Lai et al. (2010) studied healthy older adults whose mean age was in a range similar to that of our controls [Lai et al.: 64.79 (7.75); our controls: 64.70 (4.24)]. However, their controls had more years of education than our controls [Lai et al.: 9.70 (4.16); our controls: 5.30 (4.03)]. Considering that Lai et al. used auditory stimulation which is known to lead to faster P300 latencies in adults than visual stimulation (see Squires et al., 1977; Johnson Jr., 1989), we could argue that the control participants of these two studies show very similar P300 latencies [Lai et al.: 404.00 (32.14); our controls: 445.13 (27.0)]. These groups were recruited in countries with a very different socio-cultural background thus suggesting that the P300 component might not vary significantly as functions of the background education. This renders the analysis of the P300 a very useful tool for assessment across cultures and countries.

A potential account for the lack of sensitivity of the latency of the P300 component to the background education could be found at a cognitive level. This ERP provides a measure of the time taken for stimulus evaluation and classification but is relatively independent of response selection and execution processes (Kutas et al., 1977; McCarthy and Donchin, 1981). Hence, the P300 taxes early attentional processes involved in memory functions which operate at a low demand level. This may explain why the specificity of the latency of the P300 in the small sample of healthy older adults, MCI and AD patients assessed here was 100% as healthy older adults with low cognitive reserve do not show decline in these early cognitive processes. Tests such as free recall of word lists do pose greater demands both on cognitive processing and on the background education (Ardila et al., 1994). As AD impacts on early top-down attentional mechanisms from its preclinical stages (Rapp and Reischies, 2005; Li et al., 2011; Olichney et al., 2011), such an assessment would identify early cognitive decline that is not accounted for by limited cognitive reserves. This evidence together with our findings suggest that the combined analysis of the P300 and sensitive neuropsychological variables would yield more reliable assessment methods which can tackle important challenges in current clinical settings in a globalized world. If we also consider the low cost of this technique compared to, neuroimaging techniques (fMRI, PET or SPECT), the use of these combined tools in the evaluation of AD and MCI appears to be feasible. Nevertheless, the diagnosis of dementia is a clinical one and therefore the use of the P300 for such purposes will always depend on how well its outcomes fit within the general clinical assessment.

One potential criticism to our study is the small sample size. To address this issue, we have undertaken a number of *a priori* and *post hoc* analyses to show that this should not limit the validity of the results presented here. Despite the relatively small sample used in this study, the results were statistically significant and suggest that patients with MCI could be better classified if neuropsychological and P300 variables are considered together. For example the sensitivity for this group increased from 80% for the Recall of Word List and 80% for the P300 Latency, to 96% when the results of these tests were jointly analyzed. The follow up data from the four MCI patients also suggest that when this clinical category is accompanied by abnormal P300 parameters, the likelihood of progressing toward AD is higher than when normal P300 parameters are observed at baseline. Future longitudinal studies should further investigate this preliminary observation. Our data fit recent suggestions of using multiple biomarkers to increase the sensitivity and specificity of detection methods for neurodegenerative dementias in general and AD in particular (Rachakonda et al., 2004). While the combined analysis of the latency of the P300 and memory for word lists considerably boosted the sensitivity of the assessment method, it did not impact to the same extent on the specificity which, although high (80%), was kept at the level of the neuropsychological variable. The combined use of measures from different levels (neuropsychology and neurophysiology) implies a more adequate integrated approach to AD and MCI research (Kuljis, 2009). For example, clinicians could focus on the combined approach for detection (i.e., sensitivity) and give more weight to the P300 latency in the separation of healthy from pathological aging (i.e., specificity).

Moreover, although high density arrays are currently available, we chose for this study only two recording sites (i.e., Pz and Fz). These have been suggested as the locations where the P300 component shows its optimal parameters (i.e., latency and amplitude; Osawa, 2001). This very simple, easy to apply, and inexpensive method proved sufficient and would allow adequate recording and later comparison with other neuropsychological variables in any clinical research settings. Finally, we have identified significant P300 changes and poor memory performance in a small group of MCI patients who, according to their profile, presented with the amnestic form of cognitive impairment. This is known to be the form of MCI that most commonly leads to AD (Bozoki et al., 2001; Lopez, 2003; Lopez et al., 2003; Fields et al., 2011). However, it is known that not all MCI patients will eventually convert to AD (Lonie et al., 2010). Although the P300 parameters deteriorate as AD progresses (Ball et al., 1989), this component has not been extensively used to monitor longitudinally MCI or AD patients. Therefore, future studies should address whether the combined approach proposed here could help predict MCI to AD conversion thus permitting its use as a cognitive/functional biomarker for AD.

## Conclusion

We have combined the analysis of the P300 and standard neuropsychological variables to assess a sample of patients with MCI and AD taken from a Latin American population which has a socio-demographic structure typical of low-income countries and which had not been assessed before using this methodological approach. We have found that this combined approach can provide valuable information for the detection and evaluation of patients with MCI and AD. Our preliminary findings suggest that in populations with low socioeconomic and educational levels, the combined use of these techniques may offer a very useful method for the preclinical assessment of AD. Our results provide a platform and justification to employ more resources to convert P300 and related parameters into an accepted biological marker for AD. This would allow the definition of cut-off values which can help in the distinction between normal and pathological aging (e.g., indicators of neurodegeneration). Moreover, these norms would permit an easy, inexpensive, and objective diagnosis as well as longitudinal assessment of larger samples of MCI patients.

## Conflict of Interest Statement

The authors declare that the research was conducted in the absence of any commercial or financial relationships that could be construed as a potential conflict of interest.

## Appendix

**Table A1:** **The table shows the data corresponding to the seven reassessed participants**.

Initials	Initial status	Current status	Lat P3-Fz	Amp P3-Fz	Lat P3-Pz	Amp P3-Pz	Global cognition	Memory			Attention	Fluency	Daily life activities
							MMSE	Word list (immediate recall)	Word list (delayed recall)	Rey figure (recall)	TMT	Verbal fluency (animals)	Lawton
Cut-off values (Mean ± 2SD)			453.08	3.84	472.13	3.95	% Change	16.01	5.02	2.63	218.37	10.7	% Change
LAM	MCI	Converted to AD	564.47	3.24	558.47	3.17	10% Drop	13.00	4.00	0.00	198.00	14.00	43% Drop
MAP	MCI	Converted to AD	616.94	4.56	600.45	6.40	20% Drop	6.00	3.00	0.00	*	7.00	43% Drop
MCG	MCI	Returned to normal	416.04	9.27	437.04	10.05	29% Increase	20.00	10.00	11.50	96.00	13.00	25% Increase
BPT	MCI	Uncertain	435.53	4.19	441.53	4.61	30% Increase	18.00	8.00	11.00	98.00	19.00	43% Drop
EFR	Control	Continue healthy	441.53	5.53	447.53	9.25	3% Increase	13.00	6.00	11.00	151.00	13.00	No change
OTS	Control	Continue healthy	435.53	3.14	464.02	5.37	6% Increase	18.00	6.00	3.00	149.00	13.00	No change
MRC	Control	Continue healthy	465.52	7.79	488.00	5.03	3% Increase	15.00	5.00	11.00	93.00	20.00	No change

**Could not complete the assessment; Cut-off values: were obtained from the norms corresponding to the same population (Ardila et al., 1994, 2000); Lawton: Scale of Instrumental Activities of Daily Living (IADL)*.

*The P300 variables collected during the first assessment and the Neuropsychological variables collected during the second assessment are presented. In order to assist in the clinical decision, the neuropsychological data from the first and second assessment were contrasted. Scores such as the MMSE and the Lawton Instrumental Activities of Daily Living are expressed as percentage of change in the second relative to the first assessment*.
